# Iron entry in neurons and astrocytes: a link with synaptic activity

**DOI:** 10.3389/fnmol.2015.00018

**Published:** 2015-06-03

**Authors:** Franca Codazzi, Ilaria Pelizzoni, Daniele Zacchetti, Fabio Grohovaz

**Affiliations:** ^1^Vita-Salute San Raffaele UniversityMilan, Italy; ^2^Division of Neuroscience, San Raffaele Scientific Institute and UniversityMilan, Italy

**Keywords:** iron, neurons, astrocytes, oxidative stress, synapses

## Abstract

Iron plays a fundamental role in the development of the central nervous system (CNS) as well as in several neuronal functions including synaptic plasticity. Accordingly, neuronal iron supply is tightly controlled: it depends not only on transferrin-bound iron but also on non-transferrin-bound iron (NTBI), which represents a relevant quote of the iron physiologically present in the cerebrospinal fluid (CSF). Different calcium permeable channels as well as the divalent metal transporter 1 (DMT1) have been proposed to sustain NTBI entry in neurons and astrocytes even though it remains an open issue. In both cases, it emerges that the control of iron entry is tightly linked to synaptic activity. The iron-induced oxidative tone can, in physiological conditions, positively influence the calcium levels and thus the synaptic plasticity. On the other hand, an excess of iron, with the ensuing uncontrolled production of reactive oxygen species (ROS), is detrimental for neuronal survival. A protective mechanism can be played by astrocytes that, more resistant to oxidative stress, can uptake iron, thereby buffering its concentration in the synaptic environment. This competence is potentiated when astrocytes undergo activation during neuroinflammation and neurodegenerative processes. In this minireview we focus on the mechanisms responsible for NTBI entry in neurons and astrocytes and on how they can be modulated during synaptic activity. Finally, we speculate on the relevance they may have in both physiological and pathological conditions.

## Introduction

Iron transport in cells and body fluids requires the participation of specific proteins and buffering molecules to avoid precipitation of the metal ion, with the potential induction of cytotoxic effects. In the plasma, the main binding protein is transferrin (Tf), even though heme and ferritin can contribute to bind and circulate iron. In particular, iron can be transported as hemoglobin bound to haptoglobin as well as heme directly bound to hemopexin. On the other hand, ferritin iron represents only a minute amount of the circulating iron (Crichton and Charloteaux-Wauters, 1987; Brissot et al., 2012). Plasma iron concentration can exceed the total binding capacity of Tf only in conditions of severe pathological iron overload, as it occurs, for instance, in different forms of hemochromatosis (Janssen and Swinkels, 2009). Along with the transferrin-bound iron, another iron pool was identified by Hershko et al. (1978) and called non-transferrin-bound iron (NTBI). However this definition is somewhat confusing since NTBI does not include all forms of iron unbound to transferrin; for instance heme and ferritin iron are excluded (Brissot et al., 2012). Plasmatic NTBI is composed mainly by Fe(III), loosely bound to buffering molecules (mainly citrate and acetate), but also to the abundant plasma protein, albumin (Hider, 2002; Brissot et al., 2012). This pool has attracted great interest since it may represent a relevant source of iron for various cell types (e.g., in hepatocytes; Wang and Knutson, 2013) and can have a pathological relevance (e.g., in cardiomiocytes; Oudit et al., 2006).

Different is the situation of iron homeostasis in the central nervous system (CNS): although the mechanisms are still debated and somewhat controversial, a large body of evidence indicates that the iron control in CNS is virtually independent of the rest of the body. Indeed, conditions of systemic iron overload or deficiency are reported not to significantly affect the brain (Russo et al., 2004; Moos et al., 2007). Moreover, also the role and the contribution of transferrin-bound iron and NTBI to iron homeostasis appear clearly different in the two compartments. In fact, transferrin-bound iron is reported to operate with the same mechanisms described at the systemic level in neurons but not in glial cells. On the other hand, NTBI is attracting more attention in light of the various mechanisms that are potentially involved and of the possible regulation they are subjected to during synaptic activity (see next chapter). Based on this premise, we will focus our attention on NTBI, its composition and the mechanisms sustaining its uptake in brain cells.

## NTBI in the Brain

Brain shows clear differences in the control of iron homeostasis that can account for its specific physiopathological behavior. Despite the difficulty of measuring iron concentration within the cerebrospinal fluid (CSF) and brain interstitium, a value much lower than in the plasma was estimated (0.25–1 μM vs. ~20 μM). Considering that the concentration of the Tf produced in the brain (by the choroid plexuses and oligodendrocytes) is more than two orders of magnitude lower than in the plasma (i.e., ~0.2 μM vs. ~30 μM), Tf is expected to be fully saturated in the CSF. Accordingly, NTBI significantly contributes to total iron in CSF and brain interstitium even in physiological conditions (Molina et al., 1998; Moos and Morgan, 1998; Mizuno et al., 2005; Hozumi et al., 2011). Moreover, the presence of a very high concentration of ascorbate in the CSF (up to 100 times, compared to plasma; Bradbury, 1997; Lane et al., 2010), generates a reducing environment that increases the ratio Fe(II)/Fe(III) in NTBI. The expression of different ferric reductases (duodenal cytochrome B, Dcytb, and stromal cell-derived receptor 2, SDR2) further contributes to make Fe(II) the prominent form of iron in the NTBI (Tulpule et al., 2010). This is particularly relevant since Fe(II) represents the main form of iron acquirable by astrocytes, which do not have Tf receptors, but also an important source for neurons (Moos et al., 2007; Pelizzoni et al., 2011, 2013). However, NTBI has not only a relevant physiological role in the CNS but it can acquire a pathological relevance, as suggested by growing evidence that NTBI levels increase during aging as well as in several neurodegenerative disorders (Bradbury, 1997; Breuer et al., 2000; Crichton et al., 2011). From this standpoint, the NTBI can be related to the onset and the progression of neurodegenerative disorders, not necessarily related to genetic alteration of iron homeostasis. Therefore, a thorough characterization of the cellular pathways and of the molecular mechanisms controlling NTBI in the brain can open new vistas on the etiopathogenesis of many neurodegenerative diseases.

## Mechanisms of NTBI Uptake by Neurons and Astrocytes

A number of questions are still open about the mechanisms of NTBI entry in brain-derived cells: which are the pathways that operate in neurons and astrocytes? Which is their relevance in specific brain regions? Can neuronal functions and, in particular, synaptic activity, influence NTBI uptake? And, finally, can pathological conditions affect these pathways?

NTBI uptake was first proposed in cortical neurons (Cheah et al., 2006), where a role for divalent metal transporter 1 (DMT1), the main iron transporter expressed in mammalian cells (Gunshin et al., 1997), was reported. In particular, it was suggested that a calcium influx mediated by the glutamatergic N-methyl-D-aspartate receptor (NMDAR) is able to promote a positive modulation of the transporter, and thus of Fe(II) uptake, as a consequence of its interaction with Dexras1 (a member of the Ras family) via an adaptor protein. However no evidence of a direct involvement of DMT1 in the iron influx was provided; moreover, data were not obtained in physiological conditions, since the experiments were performed at a pH of 5.5. This paper thus proposes, for the first time, a link between synaptic activity and iron entry in neuronal cells. This general concept also emerged in a following study from Hidalgo’s group (Haeger et al., 2010) in which it was reported that a brief stimulation with NMDA was sufficient to promote the transcription of the isoform 1B/IRE(+) of DMT1 in hippocampal neurons; however, in this paper, iron entry was not evaluated. Of note, when DMT1-1B/IRE(+) was overexpressed in epithelial cell lines as well as in primary neurons, it was not localized in the recycling endosome, but in the late endosomes/lysosomes, and it was not involved in iron uptake (Yanatori et al., 2010; Pelizzoni et al., 2012). As an alternative, it was proposed that the Fe(II) may enter neurons directly through Ca^2+^-permeable channels and, in particular, voltage-operated calcium channels (VOCCs) and NMDARs (Pelizzoni et al., 2011). This mechanism, which might regard all VOCCs but not all Ca^2+^-permeable channels, involves the competition between Ca^2+^ and Fe(II) for the same entry pathway (Lopin et al., 2012), a condition also described in neuronal cell lines (Gaasch et al., 2007) These results are in accordance with those obtained on a model of iron-overloaded cardiomyocytes (Oudit et al., 2003), in which the L-type VOCCs represent the main mechanisms of Fe(II) entry and thus, of iron-induced cardiomyopathy. The possibility that neurons from different brain areas might differ for the mechanisms of iron entry remains open. Moreover neurons may also acquire the competence to activate specific pathways when the physiological conditions are altered (see below).

Also astrocytes show a great complexity of iron control, particularly considering that in these cells Tf receptor 1 was reported not to be expressed *in vivo* (Moos and Morgan, 2004; Jeong and David, 2006). Recent studies have proposed that cultured astrocytes are capable of importing both Fe(II) and Fe(III) and that in the presence of ascorbate, to reproduce the *in vivo* reducing conditions, iron accumulation is further enhanced (Lane et al., 2010; Tulpule et al., 2010). In both papers an involvement of DMT1 was proposed, even though additional pathways were not excluded. Overall, DMT1 has received great attention and has conditioned our general view of iron uptake. However, the* in vivo* expression of DMT1 was reported to be confined to astrocytic perivascular endfeet (Moos and Morgan, 2004). Other mechanisms, such as the zinc transporter Zip14, have been proposed for NTBI uptake in astrocytes, even though their physiological role is still to be established (Bishop et al., 2011).

More recently it was shown that, in physiological conditions, Fe(II) can enter the hippocampal astrocytes through the transient receptor potential canonical (TRPC) channels, with an irrelevant involvement of DMT1 (Pelizzoni et al., 2013). However, upon exposure to proinflammatory cytokines and ensuing activation, astrocytes expressed the DMT1-1A, i.e., the isoform localized on the apical membrane of enterocytes (Hubert and Hentze, 2002). Accordingly, the *de novo* expression of this isoform of DMT1 in activated astrocytes was proposed to account for their increased capability to uptake Fe(II), but not Fe(III) (Rathore et al., 2012; Pelizzoni et al., 2013; Urrutia et al., 2013). In light of these results, the discrepancies on the importance of DMT1 in astrocytic Fe(II) uptake might be ascribed to the high variability of the culture conditions that can promote different extents of astrocyte activation (Saura, 2007).

## Effects of NTBI on Neurons and Role of Astrocytes

We have seen that NTBI is a relevant source of iron and that various mechanisms can control its supply to neurons and astrocytes. This contribution is important to restore the pool of iron-sulphur clusters and thus to meet the elevate energy consumption caused by neuronal activity, but also to sustain specific neuronal functions, such as neurotransmitter synthesis. Moreover, NTBI can contribute to the “oxidative tone” which is important for both basal synaptic transmission and long-term potentiation (LTP; Muñoz et al., 2011). In particular, it was reported that the iron-mediated reactive oxygen species (ROS) production is required in hippocampal neurons to promote calcium release from ryanodine receptor after NMDA stimulation, an essential event for a sustained LTP. Therefore, a direct coupling between synaptic activity and iron entry can properly address these requirements. However, this is a double-edged sword in that iron entry, if not properly controlled, represents a harmful condition, because of the capability of Fe(II) to catalyze the so-called Fenton reaction, which occurs primarily at the mitochondrial level with production of the highly toxic hydroxyl radical (Halliwell, 2006). Accordingly, an increase in NTBI or a potentiation of the import mechanisms can cause high detrimental effects in neurons. A link between elevation of iron levels and neurodegenerative processes has been proposed for aging as well as in several pathological conditions (Altamura and Muckenthaler, 2009; Crichton et al., 2011). The toxic effects of iron accumulation in brain can be particularly harmful when Fe(II) enters the neurons through Ca^2+^-permeable channels since it is not controlled by the same intracellular iron level as it occurs for the Tf-mediated pathway and, possibly, also for DMT1 (Hentze et al., 2010). Moreover, the concomitant influx of iron and Ca^2+^ can greatly contribute to excitotoxicity in glutamatergic neurons (Dixon et al., 2012; Chen et al., 2013). More in general, conditions of increased synaptic activity, in the presence of elevated iron, can cause iron overload and concur to the development of cytotoxic effects (Pelizzoni et al., 2008), as it was suggested to occur in Alzheimer disease (Altamura and Muckenthaler, 2009). Also a potentiation of the mechanisms of iron import can lead up to this dangerous chain of effects. Indeed, an enhancement of DMT1 expression was reported after induction of excitotoxicity (Huang et al., 2006) or hypoxia (Wang et al., 2010) as well as in animal and cellular models of Parkinson disease (Salazar et al., 2008; Du et al., 2009).

This general framework can be further exacerbated in conditions of acute brain injuries and inflammation, which accompany several neurodegenerative disorders, since local acidification favors the reduction of Fe(III) to Fe(II) and potentiates the H+/metal-ion cotransport activity of DMT1 (Mackenzie et al., 2007).

Within this context, astrocytes also play an important role. They were described as participating in brain iron homeostasis under conditions of iron deprivation, with soluble ceruplasmin, released by the same astrocytes, supporting iron uptake in neurons, even though the mechanisms were not elucidated (Ke et al., 2006). More recently, it has been proposed that they participate in the synaptic activity also by buffering iron. Indeed, astrocytes are endowed with high detoxifying defences that make them more resistant than neurons to oxidative insults (Pelizzoni et al., 2011). Under physiological conditions, and even more in the presence of extracellular iron accumulation, these glial cells might buffer perisynaptic iron by uptaking it via TRPC, thereby actively contributing to lowering synaptic iron load. Of note, the activity of members of the TRPC family is enhanced by decreasing the extracellular pH (Semtner et al., 2007) to values comparable to those observed when exocytosis of the synaptic vesicle is evoked (Palmer et al., 2003).

Overall, it is temping to envision a model in which an increase in synaptic activity favors iron overload in neurons, a condition that is counteracted by the active iron buffering of astrocytes. This protective role is expected to be enhanced during inflammatory conditions when local acidification and astrocyte activation (Xiong et al., 2008), with *de novo* expression of the DMT1, further enhance iron buffering. This condition has been recently reported in an experimental model of multiple sclerosis, where a significant increase in DMT1 expression was observed in astrocytes around the lesions, making them more efficient in recycling iron from the sites of lesions (Zarruk et al., 2015). Of note, inflammatory conditions can cause astrocyte activation with ensuing higher protection against iron-mediated oxidative stress (Macco et al., 2013). Conversely, the expression of DMT1 in neurons, as reported in some pathological conditions, might favor iron overload and represent a concause in the neurodegenerative processes.

## Conclusions

**Figure 1:**
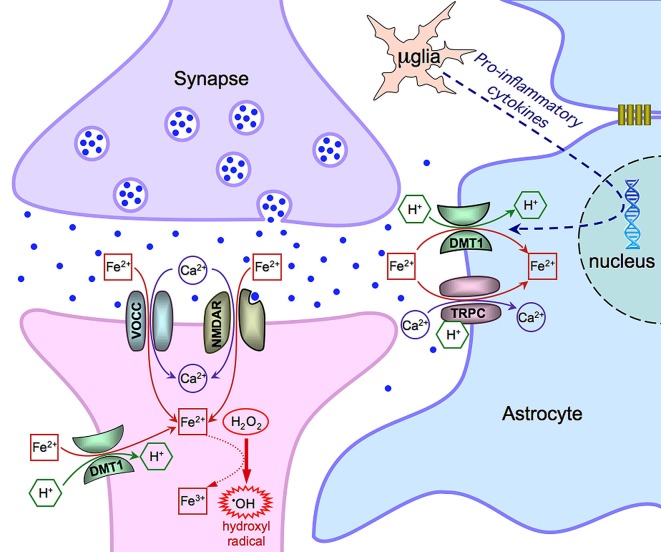
**Scheme of the main mechanisms of Fe(II) entry in neurons and astrocytes**. Notice that μglia indicates a microglia cell. See text for further details and explanations.

Data in the literature propose multiple mechanisms for iron entry in both neurons and astrocytes (summarized in Figure 1) and draw the attention to the role of the neuronal activity on iron influx. In the excitatory synapses of hippocampal neurons, iron uptake depends on the direct stimulation of NMDA receptors and indirect activation of VOCCs (by synaptic activation, back propagating action potentials or depolarization from adjacent synapses). DMT1 can have a physiological role in other brain areas, still showing a dependence on synaptic activity, or can be expressed in some pathological conditions (e.g., Parkinson disease). Within this general framework, iron uptake strongly depends on its availability that can vary with age and in some neurodegenerative diseases (Bradbury, 1997; Molina et al., 1998). In this respect, astrocytes, closely associated to neurons, operate to buffer the excess of extracellular iron. This is important under physiological conditions but, particularly, in pathological conditions when this competence is reinforced by the expression of additional mechanisms of iron uptake. Overall, this complex interplay, including the functional coupling of astrocytes through gap junctions, may help in buffering iron and in protecting neurons during neuroinflammation and neurodegenerative processes.

## Conflict of Interest Statement

The authors declare that the research was conducted in the absence of any commercial or financial relationships that could be construed as a potential conflict of interest.
